# Using intervention mapping to develop evidence-based toolkits that support workers on long-term sick leave and their managers

**DOI:** 10.1186/s12913-023-09952-0

**Published:** 2023-09-02

**Authors:** Veronica Varela-Mato, Holly Blake, Joanna Yarker, Kate Godfree, Guy Daly, Juliet Hassard, Caroline Meyer, Charlotte Kershaw, Steven Marwaha, Kristina Newman, Sean Russell, Louise Thomson, Fehmidah Munir

**Affiliations:** 1 School of Sport, Exercise and Health Sciences, Loughborough University, Loughborough, UK; 2 School of Health Sciences, University of Nottingham, Nottingham, UK; 3 NIHR Nottingham Biomedical Research Centre, Nottingham, UK; 4 Affinity Health at Work, London, UK; 5 Birkbeck, University of London, London, UK; 6 Office of the Provost, The British University in Egypt, El Sherouk City 11837, Cairo, Egypt; 7 School of Medicine, University of Nottingham, Nottingham, UK; 8 Executive Office, Warwick University, Coventry, CV4 7AL UK; 9 Warwick Medical School, University of Warwick, Coventry, UK; 10 Institute for Mental Health, School of Psychology, University of Birmingham, Birmingham, UK; 11 Psychology Department, Nottingham Trent University, Nottingham, UK; 12 Faculty of Health and Life Sciences, Coventry University, Coventry, UK

**Keywords:** Return-to-work, Long-term sickness absence, Mental health, Intervention mapping, Support, Communication

## Abstract

**Background:**

Managing long-term sickness absence is challenging in countries where employers and managers have the main responsibility to provide return to work support, particularly for workers with poor mental health. Whilst long-term sick leave and return to work frameworks and guidance exist for employers, there are currently no structured return to work protocols for employers or for their workers encompassing best practice strategies to support a positive and timely return to work outcome.

**Purpose:**

To utilise the intervention mapping (IM) protocol as a framework to develop return to work toolkits that are underpinned by relevant behaviour change theory targeting mental health to promote a positive return to work experiensce for workers on long-term sick leave.

**Methods:**

This paper provides a worked example of intervention mapping (IM) to develop an intervention through a six-step process to combine theory and evidence in the development of two toolkits – one designed for managers and one to be used by workers on long-term sick leave. As part of this process, collaborative planning techniques were used to develop the intervention. A planning group was set up, through which researchers would work alongside employer, worker, and mental health professional representatives to develop the toolkits. Additionally, feedback on the toolkits were sought from the target populations of workers and managers and from wider employer stakeholders (e.g., human resource specialists). The implementation and evaluation of the toolkits as a workplace intervention were also planned.

**Results:**

Two toolkits were designed following the six steps of intervention mapping. Feedback from the planning group (*n* = 5; psychologist, psychiatrist, person with previous experience of poor mental health, employer and charity worker) and participants (*n* = 14; employers = 3, wellbeing director = 1; human resources = 2, managers = 2, employees with previous experience of poor mental health = 5) target populations indicated that the toolkits were acceptable and much needed.

**Conclusions:**

Using IM allowed the development of an evidence-based practical intervention, whilst incorporating the views of all the impacted stakeholder groups. The feasibility and acceptability of the toolkits and their supporting intervention components, implementation process and methods of assessment will be evaluated in a feasibility pilot randomised controlled trial.

**Supplementary Information:**

The online version contains supplementary material available at 10.1186/s12913-023-09952-0.

## Background

Long-term sickness leave (LTSL) is a global challenge [1, 2] with societal and economic implications for workers, their employers, and for health and social care providers. Impacts of LTSL include social isolation, reduced workability (defined as the worker’s ability to do their job, with respect to work demands, health, and mental resources [3]; productivity [4–7] reduced wellbeing, disability pension, and a higher risk of unemployment or job termination [8]. Therefore, early intervention to support a worker’s return to work (RTW) is both cost effective for the employer and vital for the workers’ health and wellbeing.

Common mental health (MH) problems such as stress, depression and anxiety, account for 30 to 50% of all periods of sick leave at work [9] and in the United Kingdom (UK), MH problems are the third most common reason for taking time off sick [10]. This may be an underestimate due to the stigma of MH problems [11]. Mental health problems are also associated with a number of co-morbidities such as musculoskeletal pain, injuries and cardio-respiratory problems (National Institute of Care and Excellence, [12] and COVID-19 [13], which are often the focus of treatment instead of MH.

Although most workers with common MH conditions will RTW, this can be a complicated and long process [14]. Factors beyond the MH problems itself that are known to impact both RTW and ongoing work retention include lower socio-economic status, education and self-efficacy, older age, poor manager/supervisor and/or co-worker support, and inadequate workplace RTW policies and work adjustments [15–18].

Systematic reviews, including meta-analyses, on the effectiveness of RTW interventions for mental health problems have shown that psychological interventions such as work-focused cognitive behaviour therapy and work-directed solutions (e.g., managers and workers identifying work adjustments needed for returning to work) are effective in reducing sick leave and costs associated with work disability [19, 20]. Multi-component RTW interventions that target the workers poor MH and elements of their job role (e.g., changing a person's tasks or working hours) are most effective. Although the UK has well-developed frameworks for rehabilitation and RTW, there are no unique agencies coordinating the overall rehabilitation/RTW process [21]. While the National Health Service (NHS) plays a vital role in determining the fitness of a worker to continue working with some adjustments or to take sick leave it mainly focuses on the medical aspects of the process (HM Government, no date). Some employers provide occupational health support, but this is not universal, and the provision of occupational health services is inconsistent [21]. NICE [22] have highlighted that employers need to do more to support workers whilst on sick leave and when RTW, especially through the provision of better manager/supervisor support. With MH-related sickness absence being the most common as well as the most complex RTW for employers to manage, efficient and cost-effective interventions to support the process are needed.

The aim of this study is to develop an employer-led intervention to support workers with poor MH and their managers during the workers’ sickness absence and RTW process. The study is part of the Mental Health and Productivity Pilot (MHPP; https://mhpp.me/), a large research programme focussed on MH and work.

As workplace RTW interventions are complex, requiring a tailored and multi-component approach involving various stakeholders, a collaborative approach was adopted to develop the intervention using the Intervention Mapping (IM) [23] protocol. Implementation mapping has been used previously to develop RTW interventions [24–27]. However, to our knowledge, this is the first time IM is being used to develop online multicomponent RTW intervention toolkits aimed at managers (also known as supervisors) and workers, to promote manager support and worker wellbeing and RTW.

## Methods

**Fig. 1:**
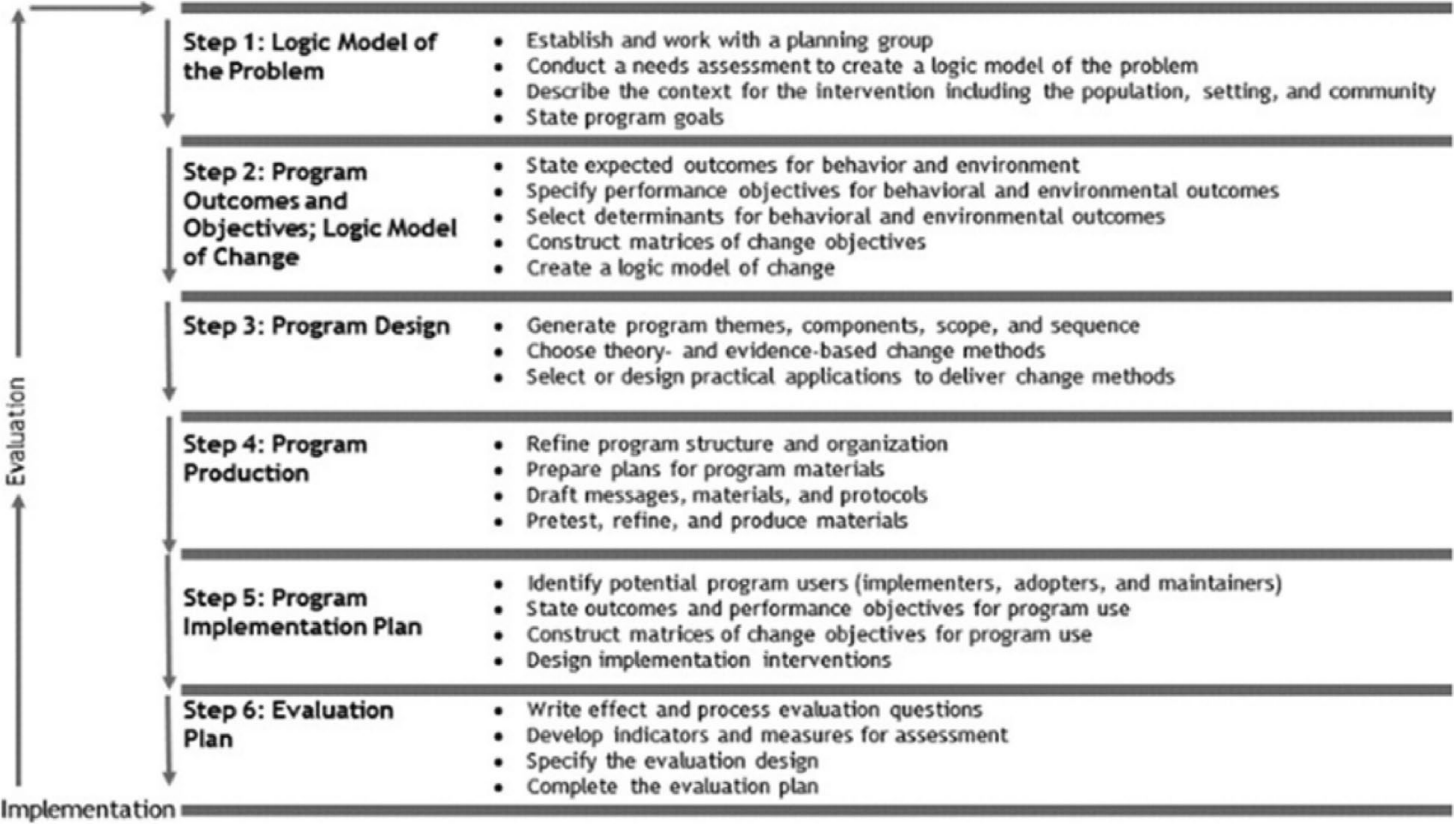
Intervention mapping framework, reproduced from Bartholomew et al (2006)

This intervention development is an early stage of a registered trial (ISRCTN registry identifier: ISRCTN90032009). Implementation mapping is based on the social ecological approach for planning and developing theory and evidence-based and behaviour change programmes. It includes both knowledge obtained from the literature and key stakeholders to develop, implement practical strategies, and evaluate an intervention [23]. The IM methodology involves 6 iterative steps of development with flexibility to revisit steps as needed (Fig. 1. Intervention mapping framework). A key aspect of IM is that it incorporates a needs assessment to include the perspectives of the target population to maximise the effectiveness of the intervention [23, 28]. To enhance this, a planning group was established to help create the RTW intervention.

The planning group consisted of five members outside of the research team including a psychologist, psychiatrist, an individual with previous long-term sick leave experience due to poor mental health, an employer, and a mental health charity worker. Some of the members were user representatives from the wider MHPP consortium. Two members of the research team (FM, VVM) with expertise in health and wellbeing and occupational psychology liaised with the planning team. The relationship between the planning group and the research team (PGRT) can be defined by the principles of the Practice Dive Approach [29]. This is when “the academic co-creators familiarise themselves with the research setting and the end-user’s needs to support the subsequent process of collaboration”. Thus, the PGRT met regularly throughout the study, either face-to-face or virtually, to gain an insight into the manager’s and employee’s needs and to seek help with the development of the toolkits. To conduct this work ethical approval was obtained from Loughborough University Ethics Sub-Committee (ref 4951).

### Step 1: needs assessment

To develop an intervention programme to locally tailor and implement the use of RTW toolkits into existing workplace settings a needs assessment was conducted including a literature review and the development of a logic model.

A rapid review of the scientific literature and professional reports/guidance was conducted to identify knowledge on factors associated with poor RTW outcomes and best practice in supporting the RTW of workers with poor mental wellbeing. Planning, conducting and data synthesis of this review followed the guidance from Khangura et al. [30] and the WHO [31]. The rapid review was carried out between May 2020 and August 2020 by FM and VVM and it included research published from 2017 onwards to supplement the earlier literature review carried out by JY and colleagues [32]. The earlier review was carried out to inform the development of a RTW toolkit aimed at small and medium-sized employers. The review was conducted from January 2007 to March 2017 and identified 15 relevant scientific articles and six professional reports/guidance (not limited to small and medium-sized employers). To bring this review up to date for this study and using the same search terms, two of the authors (FM and VVM) searched across a range of databases, including Web of Science, PubMed, Google Scholar, PsychInfo, Cochrane Library and Google search engine for the professional reports/guidance.

**Fig. 2:**
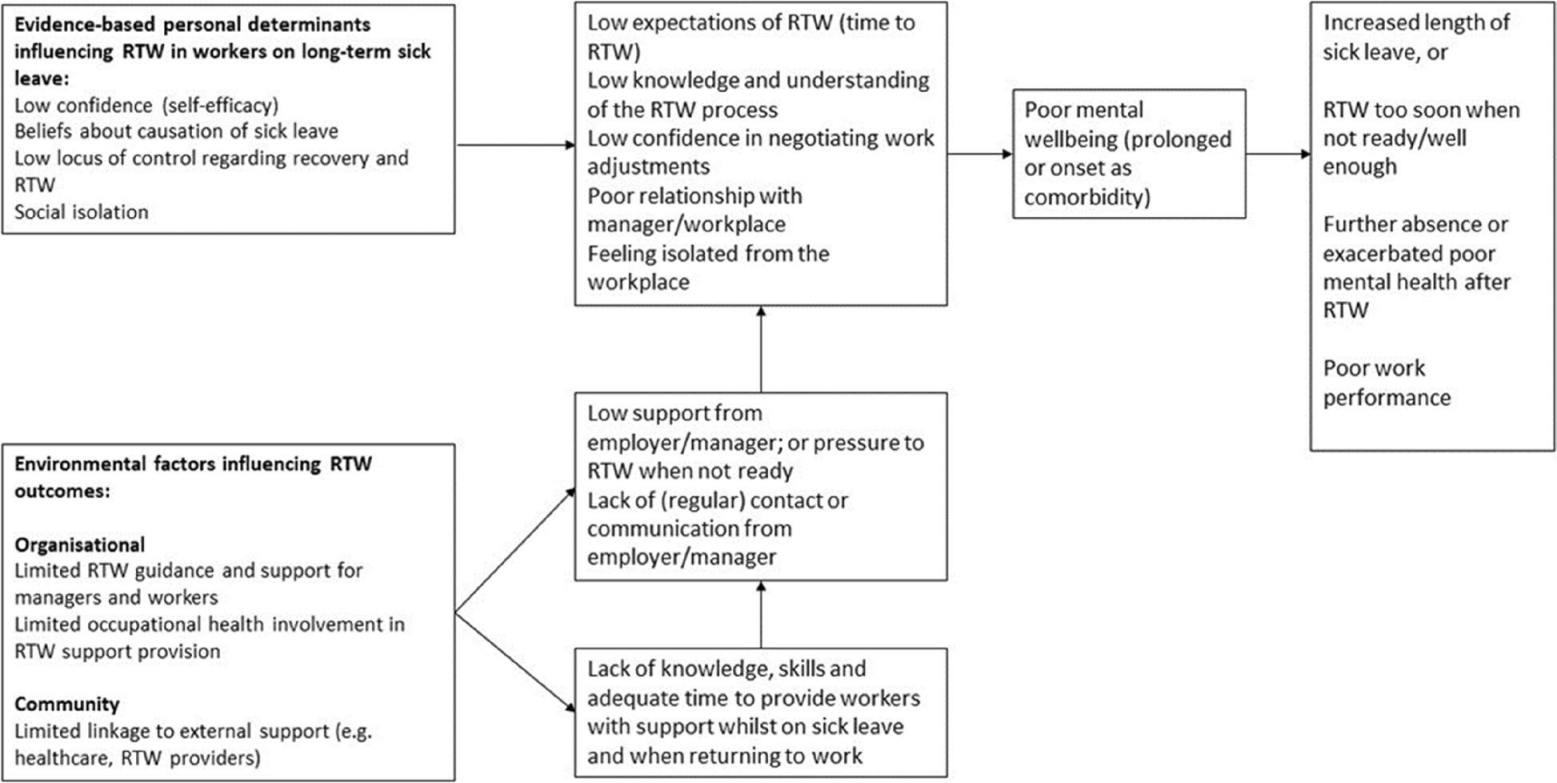
Logic model of the problem

Next, a logic model (Fig. 2) was developed to outline the problem (i.e. long term sickness absence in those with poor mental health) and its causes (e.g., low self-efficacy, lack of regular workplace contact).

### Step 2 identification of outcomes, performance objectives and change objectives

**Fig. 3:**
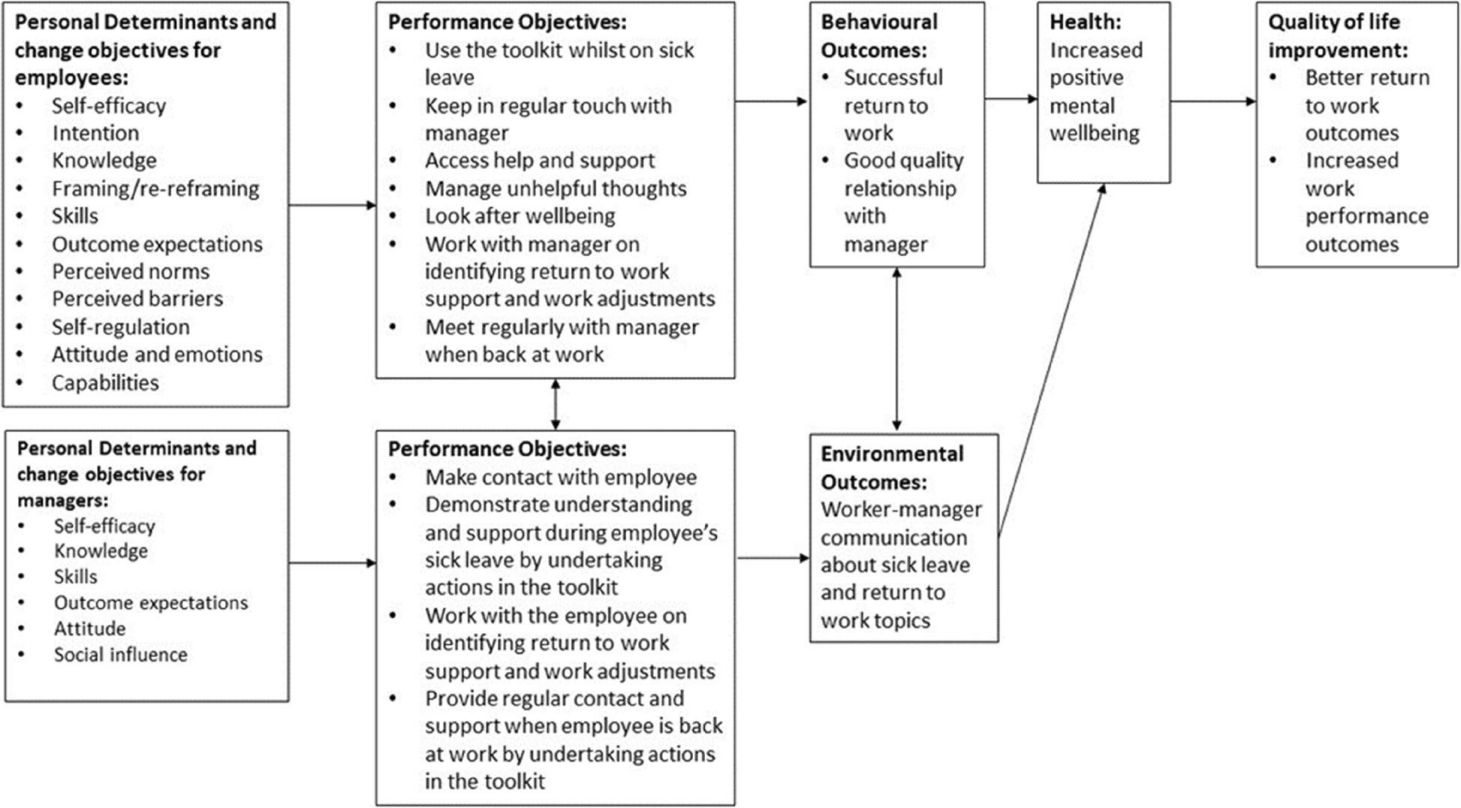
Logic model of change

In Step 2, a logic model of *change* was developed (Fig. 3) using the information from Step 1. The expected behavioural outcomes in the target groups (e.g., workers undertaking actions to support their return to work) and the performance objectives were specified (i.e., describing what is required of the target group to perform the behavioural outcomes). Behaviour change matrices were developed to capture each of the performance objectives, their change objective (e.g., the change in behaviour required to achieve the performance objective) and their theoretical determinants (factors expected to influence behaviour). For example, if a performance objective is for managers to ‘contact a worker on sick leave’, a change objective might be ‘to know what to say to their worker when contacting them’ and an appropriate theoretical determinant may be self-efficacy [33] (i.e., having the confidence in their ability to make contact and in knowing what to say). A specific description of these can be found in Additional files 2, 3, 4 and 5.

### Step 3: theory-based intervention methods and practical applications

Step 3 involved identifying suitable theoretical change methods to change behaviour from our stated change objectives (Additional file 6). These were then translated into practical approaches for the toolkits for the target groups (the worker and their manager) and chosen through examining the relevant literature, recent best practice guidelines and discussions with the planning group. Once the theoretical change methods were identified, the researchers revised the components of the logic model in step 2. A summary of the theories and their determinants can be found in Table 5 (Additional file 6).

### Step 4: intervention programme production

Step 4 comprised the development of the intervention materials and study protocol. This included defining the scope and delivery of the intervention as online toolkits. First, drafts of the toolkit content and layout were created. Using principles of user-centred design (pilot and usability testing) as used in Blake et al. [34], both toolkits were initially tested by the five planning group members, to ensure completeness, user-friendly design, and readability [35], that the resources support behaviour change, and the online toolkits are clear in scope, clarity, and presentation [36].

In addition to the feedback provided by the planning team (*n* = 5), a further 14 people were recruited as research participants representing the target group of employers (*n* = 3, from small and medium sized enterprises; 2 males), health, safety and wellbeing director (*n* = 1, from large enterprise; male), human resources (HR) business partners (*n* = 2, from medium and large enterprises; 1 female), managers (*n* = 3, from large enterprises; 2 female), and office-based and manual workers with a previous spell of LTSL related to poor mental health (*n* = 5; 2 females) to give feedback on the toolkits (Table 1). Participants were recruited via the MHPP network and existing contacts and consented prior to participation. Of these, participating managers were invited to review the manager toolkit and those with previous sick leave experience were invited to review the worker toolkit. All other participants were invited to review both toolkits. Toolkits were reviewed in paper format or an online Microsoft Word document. Participants were encouraged to write their feedback on the toolkits (e.g., comments, suggestions, amendments etc.). After a two-week timeframe to review the toolkits, semi-structured interviews (interview guides, available upon request) were conducted with each participant to discuss the content and context, presentation, clarity, usability, and functionality of the toolkits. Managers and HR participants we also asked how useful the toolkits were alongside their existing RTW guidance and practices. Interviews were digitally recorded, transcribed and themes developed using the deductive method of thematic analysis (VVM and FM), where themes were already preconceived based upon the interview schedule and existing knowledge [37]. Qualitative free text responses were then coded and narratively reported.

**Table 1:** Initial feedback on toolkit by planning group and participants who agree/strongly agree (*n* = 19)

Toolkit	Relevant Content (scope) n (%) agree	Easy to understand n (%) agree	Easy to use n (%) agree	Easy to navigate (functionality) n (%) agree	Appropriate length n (%) agree
^a^Worker (*n* = 16)	16 (100)	14 (88.0)	14 (88.0)	14 (88.0)	13 (81.3)
^a^Manager (*n* = 14)	14 (100)	12 (86.0)	11 (76.0)	14 (100)	11 (76.0)

^a^A total of 19 people reviewed the toolkits, with 11 reviewing both toolkits

The process of seeking and collating feedback was dynamic with multiple reviews and revisions made iteratively, until consensus was reached on the final versions of the toolkits. At each iteration, the performance objectives, change objectives and practical strategies for behaviour change were revisited and refined as needed. The research team then developed the protocol for the intervention study to test the toolkits.

### Step 5: programme implementation plan

The wider research team with input from the planning group developed an implementation plan for the toolkits. The group developed practical strategies at the individual and organisational level to maximise access and use of the toolkits as a real-world intervention [23]. This included identifying a) key stakeholders who would need to be involved to enable workers and their managers to be recruited into the study; b) what training the stakeholders will need and how this will be delivered, c) what resources stakeholders will need to identify workers and their managers for recruitment; and d) who will consent workers and managers into the study.

### Step 6: evaluation plan

In the final step of the IM protocol an evaluation plan was developed to assess the feasibility and acceptability of the intervention. This included deciding on how and when evaluation data would be collected, and which process evaluation frameworks would be most appropriate for the study.

## Results

### Step 1: needs assessment

#### Rapid review of the literature

This scientific review identified seven intervention or employer-led programmes [38–44], four intervention protocols [2, 45–47] and five systematic or scoping reviews on the effectiveness of RTW interventions for mental health [16, 48–51] (Additional file 1). Taking together the 15 scientific articles identified from the earlier review by Yarker and colleagues [32] and the 16 scientific articles from this rapid review, indicate that work-focused cognitive behavioural therapy (W-CBT; (e.g., problem-solving work issues) offered early into the treatment results in a faster partial RTW than regular cognitive behavioural therapy (R-CBT) without a focus on work [e.g., 41,43]. Early contact and regular communication between workers and their workplace were found to also support workers’ health and wellbeing and an earlier RTW [51]. Furthermore, work adjustments such as changes to work schedule, job task modifications, job role and work environment were also found to be important in RTW outcomes [42]. These findings align with existing systematic reviews [9, 20].

Communication-based RTW interventions were reported to lead to meaningful behaviour change for employers and managers [52]. Whilst online interventions for RTW are still in their infancy, studies show they are not only effective but are also acceptable to organisations [53, 54]. The gaps highlighted in these studies include a lack of training and upskilling of employers [51], and a lack of strong organisational culture of joint responsibility between employer and employee [16]. These may have hindered the effective implementation and sustainability of some RTW interventions.

Professional reports and guidance from the UK’s more recent NICE [12] recommend that organisations develop policies and procedures that support their worker’s health and wellbeing whilst on sick leave and when RTW and that employers make early and positive contact with their workers on LTSL to make them feel valued, supported, and confident to RTW. However, to our knowledge at the time, there were no interventions or strategies to test these guidelines in practice.

The PGRT used the review results and the experience and expertise of the group to develop a logic model of the problem (see Fig. 2 in the supplements). The logic model outlined the factors that cause or prolong poor mental wellbeing whilst on sick leave, and the problems this subsequently causes for RTW.

### Step 2 identification of outcomes, performance objectives and change objectives

The PGRT agreed the expected outcome of the intervention is the successful RTW (returning to normal contracted hours or reduced hours) of the worker who had been on LTSL. ‘Success’ was defined as taking fewer days off work compared to a control group throughout the organisation’s involvement in the trial (i.e., 12 months). However, RTW can be defined as both a process and an outcome related to when an individual returns to work after sick leave [53]; as RTW interventions can improve feelings of confidence and empowerment in the worker when returning to work [52]. RTW interventions also improve wellbeing and workability after a worker has come back to work, through the mechanism of the manager providing regular communication and support whilst the worker is on sick leave [18, 32]. Therefore, informed by our discussions and the review findings, the list of performance objectives and behaviour change matrix were constructed by the research team accounting for the complexity of RTW as a process as well as an outcome (Additional files 2,3, 4 and 5). The PGRT agreed the intervention that the RTW intervention should reflect current evidence-based best practice recommendation identified from the review [12] and comprised of online toolkits—one aimed at the manager and the other aimed at the worker. This would provide both parties the opportunity to monitor and record key actions during the sick leave and RTW process. Next, the toolkits were designed to take participants through a three-step process: 1) managing sick leave, 2) preparing to RTW, and 3) being back at work. The content for these steps were created separately for the manager and the worker RTW toolkit and the theoretical determinants involved in changing behaviour were identified (See Supplementary file).

In short, the main theoretical determinants for the change objectives for workers (see step 3 for details and Fig. 2) were intention (change objective example: formulating and implementing commitment to use toolkit), knowledge (e.g., describing resources and support needs whilst on sick leave), self-efficacy (e.g., feeling confident in being able to monitor and take action for own wellbeing and support needs), attitude (e.g., feeling positive about re-evaluating thoughts and reframing if necessary), skills (e.g., demonstrating ability to undertake actions identified), perceived norms (e.g., recognising that nowadays workers are being encouraged to take an active part in their care), and outcome expectations (e.g., expecting that using the toolkit will improve wellbeing and relationship with the workplace). To further support the performance objectives for the workers, we identified the need for workplace health coaching (WHC) (see step 2 in the methods) to support engagement with the intervention by encouraging the worker to set goals, solve problems by undertaking actions and to self-reflect on their thoughts and actions. Each employee participant would receive three coaching sessions: at intervention start, 2 months and 3 months (1 month before study participation end). Performance objectives were therefore also identified for the (WHC) with theoretical determinants (described fully in step 3 and Tables 2 and 5) such as attitude (e.g., expressing feelings about the benefits of the RTW toolkit), self-efficacy (e.g., expressing confidence in actively listening to worker and confidence in helping worker to identify appropriate goals and actions), and skills (e.g., demonstrating actively listening to worker’s concerns and ideas around goal setting) identified as main theoretical determinants. For managers, knowledge (e.g., describing ways to express support to the worker on sick leave), self-efficacy (e.g., expressing confidence in contacting the worker on sick leave), skills (e.g., demonstrating ability to communicate regularly with the worker), and outcome expectations (e.g., expecting that communicating with worker regularly will lead to a positive experience for the worker) were the main theoretical determinants for the change objectives (Additional files 4 and 6). Additionally, managers would be supported with the provision of e-learning training (see step 2 in the methods) to support their confidence to have conversations about mental health and help their worker(s) during the RTW process.

For workers and managers, a key performance objective is good regular communication – listening with awareness or empathy, being open to exchanging information and ideas, and using a friendly approach in key conversations. To facilitate this, improving communication skills is directly or indirectly (e.g., undertaking actions) reflected in several of the key change objectives.

Involvement of relevant stakeholders, such as HR staff, was deemed vital for those on LTSL to help them achieve the behavioural outcomes of the intervention [55, 56]. Thus, performance objectives, change objectives and their theoretical determinants (Tables 4 and 5) were also identified for this group to ensure that current sick leave policy and RTW guidance and procedures would not be obstacles for managers and workers in achieving the expected outcomes (see Supplementary file).

### Step 3: theory-based intervention methods and practical applications

Several theories were identified as being relevant to the active ingredients of the intervention: Communication Accommodation Theory (CAT) [57, 58], Implementation Intentions Theory [59], and Conservation of Resources (CoR) Theory [60] were selected for the manager’s toolkit. For the worker, Implementation Intentions [61], Transtheoretical Model of Change (TMC) [62] and the Social Cognitive Theory (SCT) [33] were most relevant. The cognitive behavioural elements in the toolkit were also informed by principles of problem-solving and cognitive behavioural approaches [63–65]. The theories were selected as they have established theoretical change methods (Additional file 6).

Through several planning group meetings, theoretical change methodologies were translated into specific practical strategies to influence behaviour and environmental changes (Additional files 2, 3, 4, 5 and 6). The strategies were decided based on what would likely be acceptable and feasible, given the target population and according to previous successful strategies developed by JY and FM [32]. Key practical strategies included providing checklists for workers and managers to monitor and record actions they have taken and actions to take within a self-specified timeframe (e.g., finding the organisation’s sickness absence policy before contacting the worker/manager). This strategy was agreed by the research team and planning group early-on in the IM process to reduce the cognitive burden on both workers and their managers. Other strategies included using an adapted version of Ellis’s ABCDE framework for changing irrational thoughts [66]. This is typically used in CBT including self-CBT. The framework was included as a worksheet in the worker’s toolkit and supported with information on its use and effectiveness. The use of the worksheet would be supported by the study researchers (VVM, KG, FM) trained in workplace health coaching (www.centreforcoaching.com) as part of their research role. Other strategies included worksheets on goal setting, action planning and self-reflection using SMART goals [67], for the worker to focus on their health and wellbeing and in identifying relevant support. Worksheets on solution-focused problem-solving using the GROW model [68], were also included to support the worker when preparing and planning to RTW. The worksheets consisted of guided questions designed to generate, evaluate, and reflect on goals, options, and actions. Both worksheets were supported by a session with the workplace health coach.

Practical strategies to influence behaviour change in managers included an e-learning course. Online approaches to workplace training allow for greater flexibility in learning and increase workplace training capability [69]. The rapid shift to online learning during the coronavirus (COVID-19) pandemic [70] provided an opportunity for a more contemporary approach to the delivery of manager’s training.

### Step 4: intervention program production

In step 4, the planning group (*n* = 5) and 14 research participants representing the employers, managers, and worker target groups provided feedback on the toolkits (Table 1). Their responses were analysed following the principles of deductive thematic analysis [37]. First, a template was created with our predefined themes: content and context, presentation, clarity, usability, and functionality of the toolkits. As the transcripts from the14 research participants represented different stakeholder groups from a range of sectors and sizes, VVM and FM separately analysed each transcript against these themes and compared their findings. There was overall good agreement between the two authors with minor disagreements resolved. The results were written up by VVM and checked by FM. Toolkit findings are presented using predefined themes outlined in the method section.

All 14 research participants described their organisation’s sick leave and RTW processes. Employer stakeholders and managers felt that whilst ‘fairly good’ sick leave and RTW processes existed in their organisation, more guidance and support is needed to enable all managers to use consistent practices to successfully support workers on LTSL. One employer stakeholder also felt more guidance was needed in how to discuss MH when someone was off on sick leave:

“Although businesses tend to talk a lot about mental health, this tends to be missed when discussing RTW” (female HR director).

Workers also expressed similar opinions:

“We need better understanding of return-to-work processes … when you are suffering, you know, you are not all there, it’s hard to think how that [process] could help you” (male worker).

*Toolkit feedback: *Content and context: A common agreement between the planning group and research participants suggested that the content of the toolkits was relevant, comprehensive and novel, with no existing comparable alternative at the time of the study. One participant worker with previous LTSL experience summed up the gap the toolkits would fill:

“I’ve looked at other ones, like those you can get through your employer and [private healthcare] and theirs isn’t really impressive – they’re pretty bad really, whereas this one is really comprehensive”. (Male manual worker).

All agreed that the MH focus of the toolkit was appropriate, had *“lots of human elements to and lots of links to helping people out”* (female office worker), and encouraged thinking about the wellbeing of others:

“Found really useful the definition of mental health issues. As soon as I read it, I realised that I had someone within my staff that I need to pay attention to”. (Health and Safety Manager).

Only one employer (who was a HR participant) felt the content was too slanted towards those on sick leave for poor MH and there should be an acknowledgement that those with a physical condition could develop poor MH whilst on sick leave as “*conditions often co-exist—it is very common for mental health issues to be triggered as a result of long-term health issues”.*

Presentation: Most agreed that the toolkits were easy to navigate because they were structured into three steps of managing: a) initial sick leave, b) preparing for RTW, and c) managing being back at work. This made it easy to navigate back and forth quite easily as one participant stated, *“the step-by-step approach would make the return process easier”* (Male office worker).

Usability: Most agreed that the checklists and worksheets were extremely useful. For the participant workers, the worksheets on ‘Thoughts about Work’ and ‘Your Support Network and Social Connections’ were most valuable as *“everyone could relate to at least one thought”* (female office worker) in the first worksheet, and the second one *“actively encourages someone feeling low to seek out a support network”* (female office worker).

Functionality: There were mixed views about the length of the toolkit. Whilst most agreed that the toolkits were easy to use, members of the planning group highlighted that the complexity of the language in some places made it difficult at times to easily understand what was being suggested and it was recommended that the language was made simpler. Other suggestions for improvement can be found in Table 2.

**Table 2:** Implementation of suggested improvements to the toolkits provided by the planning group (*n* = 5) and the target participants (*n* = 14)

**Planning group suggestions**
a. Combined some of the RTW planning checklists in both toolkits to streamline and build progression in the activities undertaken
b. Added information to contextualise the signs and symptoms of poor mental health in a workplace environment (manager and worker toolkit)
c. Made it clearer that the toolkits are linked and mirror each other in the three steps
d. Included further links and resources that the planning group were aware of
**Target participants**
a. Added more visuals to break up the written text
b. Added flow diagrams to summarise the steps of the toolkits

**Fig. 4:**
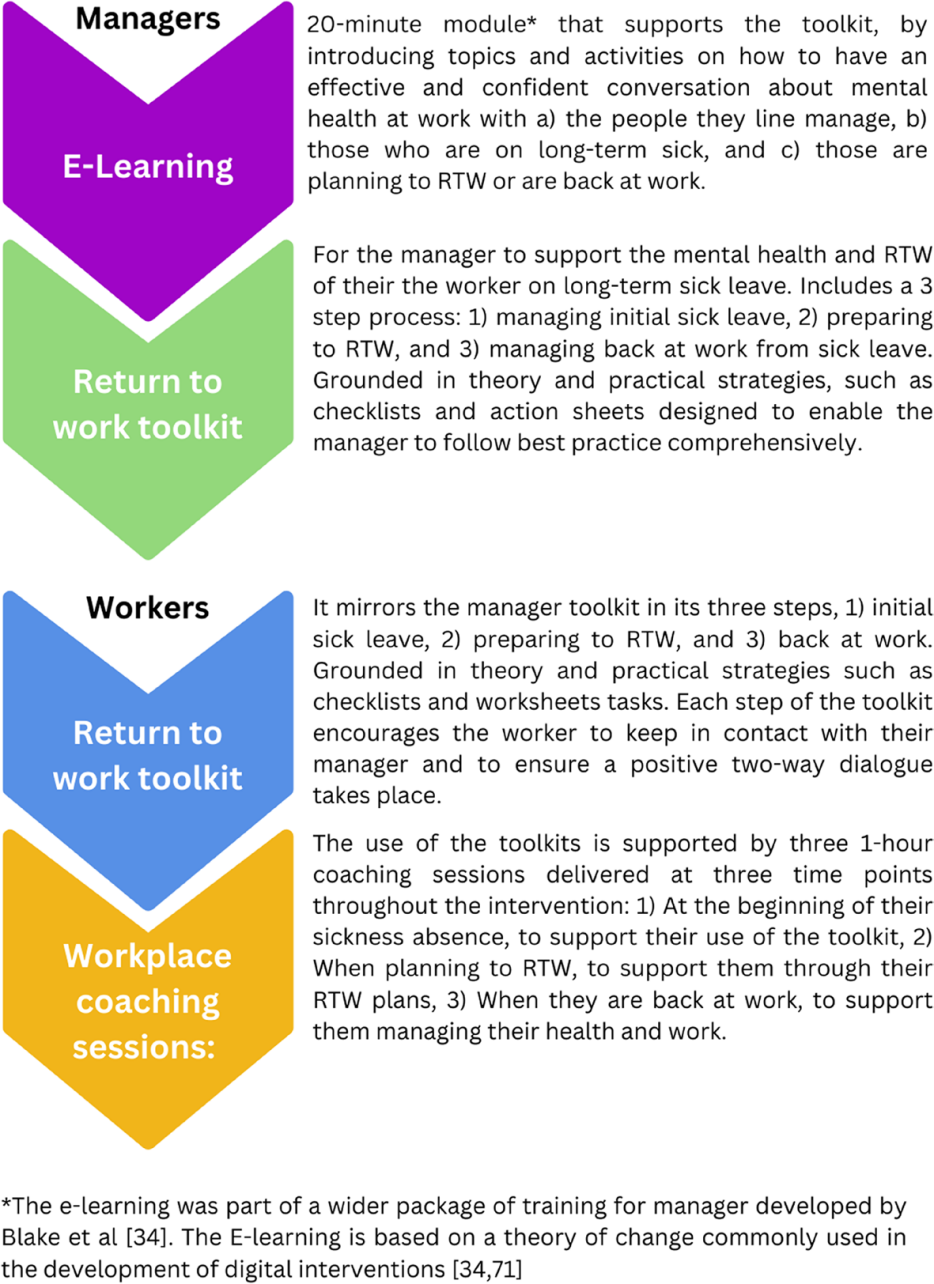
PROWORK toolkit content

Further revisions were made to the toolkits which were reviewed again by the planning group and two participants (one employer and one worker), who made further suggestions to the language and visual aids iteratively, until consensus was reached on final versions of the toolkits. Once the content of the toolkits was developed, a web design team mapped the structure and content of the toolkits onto a website (https://institutemh.org.uk/mhpp). The website was designed so the toolkits could be navigated and downloaded easily as a whole, in steps or for each individual checklist or worksheet. The planning group and the research participants gave feedback on the look, navigation and ease of use which were addressed by the web team. was recognised that a timescale for the intervention use could not be set as individuals’ length of long-term sick would vary and they would need to be supported at a pace appropriate to their situation and circumstances. However, information on the use of the intervention materials and length of an individual’s LTSL would be monitored through the study’s process and research evaluation measures. The final intervention content is shown in Fig. 4 (PROWORK: PROmoting a Sustainable and Health Return to WORK intervention content).

### Step 5: programme implementation plan

The implementation of this intervention is fully described elsewhere [71] and reports the protocol for a feasibility pilot randomised controlled trial (RCT) to assess the feasibility and acceptability of both the toolkits and planned route of recruitment. The research team developed the identity of the intervention as being named PROWORK (PROmoting a Sustainable and Healthy Return to WORK). Workers on LTSL will be recruited from 2 to 8 weeks of their sick leave. This time frame was selected as workers require a note from their doctor, known as a fit note, for any sick leave longer than 7 days. Recruitment was up to eight weeks to allow flexibility with organisations’ own sick leave reporting systems. The PGRT agreed that eligibility criteria for participation would be sickness absence associated with either poor mental wellbeing or where poor mental wellbeing may be a comorbidity [72] as RTW intervention involving the latter was of interest to NICE [12]. The study was registered on ISRCTN (ISRCTN90032009).

Organisations will be recruited to participate in the study and key stakeholders (e.g., human resources staff) will be trained to identify workers on LTSL based on their fit note and study inclusion criteria. Training will be delivered online by the research team and supported by written materials for the employer/HR team. The employer/HR staff will invite the manager and the worker on sick leave to take part in the study. Consenting of the manager and the worker will be carried out by the research team. Worker participants will be encouraged by the researchers (through the coaching sessions and written materials) to use the toolkit up to six weeks after their RTW.

As each organisation may have different sickness absence reporting systems, the PGRT agreed to work closely with the HR contact to ensure they were confident in monitoring their sickness absence reporting systems monthly; and applying the eligibility criteria.

### Step 6: evaluation plan

An evaluation plan was created to assess the feasibility and acceptability of the intervention toolkits and to address the key uncertainties in designing a definitive trial. In brief, organisations will be randomised to the control, or intervention group and trial evaluation data including the RTW outcome (still on sick leave or back at work)will be collected at baseline, 2 months and 4 months (end of study) Full details are available elsewhere [72].

The process evaluation will be informed by the Implementation Outcome Framework (IOF) [73–75] and the Theoretical Domains Framework (TDF) [74, 76] with data collected on recruitment, retention and intervention adherence. Feedback on the intervention acceptability will be collected on completion of the trial using semi-structured interviews with workers, managers, and HR colleagues. These will be supplemented with thoughts and observations about the organisation’s procedures and implementation approach and notes from the monthly calls between the research team and the organisation’s HR contact. Collectively, this information will provide insight into how participants and organisations experienced the intervention, including barriers and facilitators to implementation. Research outcome measures will also be collected at baseline, 2 months, and 4 months with the primary outcome of interest being the total number of days of sick leave until partial/full RTW as a result of intervention, to inform the planning of a larger trial.

## Discussion

This article describes the systematic development of a comprehensive RTW intervention programme for workers on LTSL either due to poor MH or another health reason where poor MH is a known comorbidity. This is the first study to outline the design of two RTW toolkits, one for managers and one for workers, using behaviour change theories and strategies, mirror conversation techniques and training to support worker’s wellbeing whilst on sick leave and to promote a sustainable RTW using an IM approach. Designing and implementing a RTW programme is a complex process due to the multi-faceted nature of RTW, particularly if the person is off-sick due to poor MH. Thus, PROWORK aims to encourage positive and early communication between the worker and their workplace.

Prior knowledge and expertise of IM by the principal investigator (FM) [24, 69, 77], enhanced the use of IM in this study. For example, to guide the needs assessment in step 1, moving flexibly between the steps with ease and including a pilot and usability assessment of the toolkit contents in step 4. This resulted in the development of a clearly justified and structured intervention. The intervention has a strong theoretical background and is underpinned by behaviour change techniques that supports behaviour change in the target groups. Our pragmatic approach to toolkit development and engagement of employers will enable managers to use the toolkit alongside existing sickness absence and RTW policies and practices. This is particularly vital in any real-world trial where intervention efficacy has not yet been established and attempting to change or adjust existing policies and practices at the development and feasibility testing stage of a study would not be sensible.

To our knowledge, in comparison to other IM studies for RTW interventions [78, 79] this is the first time IM approach that has been applied for a RTW intervention to be used directly by employers, managers and the workers themselves, without the involvement of healthcare professionals (HCP), RTW coordinators or providers (the provision of three coaching sessions in this intervention is provided to facilitate intervention engagement). This is important given that traditional RTW support, mostly offered by HCP or providers, tends to be inconsistent and does not always reach people with poor mental wellbeing [80]. Therefore, this RTW intervention provides a model to bridge the gap between the latest evidence, needs of those with poor MH in LTSL and practice in the workplace, by offering a streamlined approach for employers to support their employees’ mental wellbeing more effectively. This intervention also addresses a key recommendation from the UK’s NICE [12] guidelines that employers need to do more to support workers whilst on sick leave and when RTW, especially through provision of better manager support.

There are some limitations to this study. First, whilst the planning group included key stakeholders, there were no representatives from policy stakeholders and the worker representative had been on long-term sick leave more than five years ago. Despite these limitations, the wider MHPP project group were regularly consulted on the development of the toolkits. Two workshops run at the beginning of the project and interim reports were shared throughout. Members of the MHPP project group included representatives from our target population (e.g., workers with poor mental health, managers and employers) and key policy stakeholders. A second limitation of the study is the arrival of UK’s first national COVID-19 lockdown in the spring and summer of 2020 which impacted the recruitment of participants in step 4. We had hoped to recruit at least 10 participants from each target population, but this was difficult as employers focused on rapidly making changes to the way their workforce worked, including furloughing many of their staff. However, we tried to maintain stakeholder exchange by offering alternative approaches to participation according to the needs of the target population, including one-to-one conversations, emails, online group meetings. Thus, we feel that the number of participants involved in this research and the variety of contributions is sufficient to meet the aims and objectives of the study [81].

## Conclusions

This paper maps the development of a RTW intervention to support those on LTSL due to poor mental wellbeing and their managers during the RTW process. The findings from our interviews show that more support is needed during LTSL and that the toolkit could address this gap. Following the IM protocol allowed to identify the specific needs of the target population and implementation strategies to overcome local barriers within employer organisations. Results from the feasibility testing may provide further information about the delivery and uptake of the toolkits by workers and their managers and preliminary information about the effectiveness and cost-effectiveness of the intervention.

### Supplementary Information

**Additional file 1.** Review search terms and studies.

**Additional file 2.** Performance objectives, determinants and change objectives for the worker on long-term sick leave. list of performance objectives and behaviour change matrix for the worker on long-term sick leave.

**Additional file 3.** Performance objectives, determinants and change objectives for the workplace health coach. List of performance objectives and behaviour change matrix for the workplace coach.

**Additional file 4.** Performance objectives, determinants and change objectives for the manager. List of performance objectives and behaviour change matrix for the manager.

**Additional file 5.** Performance objectives, determinants and change objectives for the Human resources team. List of performance objectives and behaviour change matrix for the manager.

**Additional file 6.** Summary of selected theories. It includes a description of the theoretical change methods to change behaviour from our stated change objectives and a summary of the theories and their determinants.

## Data Availability

The datasets used and/or analyzed during the current study will be available from the corresponding author on reasonable request.

## Abbreviations

IM: Intervention mapping
RTW: Return to work
LTSL: Long-term sickness leave
WHO: World Health Organisation
UK: United Kingdom
NICE: National Institute for Health and Care Excellence
MHPP: Mental Health and Productivity Pilot
PROWORK: Promoting a sustainable return to work
CoR: Conservation of Resources
IOF: Implementation Outcome Framework
TDF: Theoretical Domains Framework
HR: Human resources
MH: Mental health
NHS: National Health Service
PGRT: Planning group and the research team
W-CBT: Work-related cognitive behavioural therapy
R-CBT: Regular cognitive behavioural therapy
WHC: Workplace health coaching
CAT: Communication Accommodation Theory
TMC: Transtheoretical Model of Change
SCT: Social Cognitive Theory
RCT: Randomised controlled trial
HCP: Healthcare professionals
