# Supplementary material for: Using intervention mapping to develop evidence-based toolkits that support workers on long-term sick leave and their managers
Source: BMC Health Serv Res. 2023 Sep 2;23:942. doi: 10.1186/s12913-023-09952-0 (PMC10474744; doi:10.1186/s12913-023-09952-0)
Supplement: Supplementary file 1 — Additional file 1. Review search terms and studies. [file 12913_2023_9952_MOESM1_ESM.docx]

**Additional file 1**

**Search terms**

Search terms for Yarker et al [33] and current study. First review search conducted for published articles, reports and guidance from 2005 to March 2017. Current searches were conducted between May and August 2020 with dates restricted to April 2017 to August 2020.

*Scientific literature*

The search used the following key words referring to mental health (“mental health,” OR “mental disorders,” OR “burnout,” “stress,” OR “work stress,” OR “affective disorders,” OR “emotional distress” OR “adjustment disorder,”) were combined in turn, using AND with terms related to roles (“employee,” OR “worker,” OR “employer,” OR “manager,” or “supervisor”) and with terms related to sickness absence and return to work, (“sick leave,” OR “Sickness absence,” OR “absenteeism,” OR “work absence,” OR “return* to work,” OR “medical leave,” OR “sick list”), and with terms referring to intervention (“intervention,” OR “program,*” OR “management,” OR “guidelines,” OR “guidance,” OR “feasibility,” OR “acceptability,” OR “trial” OR “toolkits”) until all combinations had been searched.

*Professional reports and guidance*

The search used the following key words referring to mental health (“mental health,” OR “mental disorders,” OR “burnout,” “stress,” OR “work stress,” OR “ common mental health problems”) were combined in turn, using AND with terms related to roles (“employee,” OR “worker,” OR “employer,” OR “manager,” or “supervisor”) and with terms related to sickness absence and return to work, (“sick leave,” OR “sickness absence,” OR “absenteeism,” OR “work absence,” OR “return* to work,” OR “rehabilitation”), and with terms referring to guidance, policy or practice (“guide,” OR “guidelines,” OR “guidance,” OR “programme,” OR “adjustments,” OR “practice,” “process,” OR “policies” OR “toolkits”) until all combinations had been searched. Key terms were also searched on the websites of the National Institute for Health and Care Excellence (NICE), Health and Safety Executive (HSE), Chartered Institute of Personnel and Development (CIPD), ACAS and Business in the Community.

**Earlier review by Yarker et al [33]**

This review was carried out for a project commissioned by the Department of Work and Pensions to develop return to work toolkit specifically for SMEs. The review identified the following scientific articles and professional reports/guidance identified as relevant to return to work and the **summary of articles or reports identified between January 2007 to March 2017 in the rapid review** are presented below:

| **Rapid literature review** | | |
| --- | --- | --- |
| **Reviews** | **Intervention studies/employer-led programmes** | **Intervention protocols** |
| Blank et al (2008)  Durand et al (2014)  Pomaki et al (2012)  Dewa et al (2016)  Cullen et al (2017)  van Vilsteren (2015)  Anderson et al (2012) | Alonso et al (2017)  Arends et al (2014)  Munir et al (2012)  Victor et al (2017)  Volker et al (2015)  Wåhlin et al (2012) | Pedersen et al (2014)  Poulsen et al (2017) |
| **Professional reports/guidance** | | |
| British Occupational Health Foundation (n.d)  IRRST (2014)  Mental Health Foundation (n.d.)  NICE (2009a)  NICE (2009b)  Scottish Government (2016) | | |

Summary of from the **updated review** **articles or reports identified between April 2017 and August 2020 in the rapid review** are presented below.

| **Rapid literature review** | | |
| --- | --- | --- |
| **Reviews** | **Intervention studies/employer-led programmes** | **Intervention protocols** |
| Axén et al, 2020  Corbière et al, 2020  Boštjančič et al, 2020  Etuknwa et al, 2019  Mikkelsen et al, 2018 | Fischer et al, 2020  van de Poll et al, 2020  Bastien et al, 2019  Brenninkmeijer et al, 2019  Kenning et al, 2018  Dalgaard et al, 2017  Lokman et al, 2017 | Andersén et al, 2020  Kouvonen et al, 2019  Sikora et al, 2019  Björk et al, 2018 |
| **Professional reports/guidance** | | |
| NICE 2019  ACAS (n.d)  Mental Health Foundation (2020)  Mind Charity (n.d) | | |

**References from review outcome**

1. ACAS (n.d). Returning to work after absence. <https://www.acas.org.uk/absence-from-work/returning-to-work-after-absence>
2. Alonso S, Marco JH, Andani J. Reducing the time until psychotherapy initiation reduces sick leave duration in participants diagnosed with anxiety and mood disorders. Clin Psychol Psychother. 2017; 25(1):138-143. <https://doi.org/10.1002/cpp.2134>
3. Andersén Å, Berglund E, Anderzén I. Rehabilitation coordinator intervention versus control in psychiatric specialist care for return to work and reduced sick leave: study protocol of a randomised controlled trial. BMC Public Health. 2020;20(1):1-8. <https://doi.org/10.1186/s12889-020-8238-3>
4. Andersen MF, Nielsen KM., Brinkmann, S. Meta-synthesis of qualitative research on return to work among employees with common mental disorders. Scand J Work Environ Health. 2012;38(2) 93-104. <https://doi.org/10.5271/sjweh.3257>
5. Arends I, van der Klink JJ, van Rhenen W, de Boer MR, Bültmann, U. Prevention of recurrent sickness absence in workers with common mental disorders: results of a cluster-randomised controlled trial. Occup Environ Med. 2014; 71(1), 21-29. <https://doi.org/10.1136/oemed-2013-101412>
6. Axén I, Björk Brämberg E, Vaez M, Lundin A, Bergström G. Interventions for common mental disorders in the occupational health service: a systematic review with a narrative synthesis. Int Arch Occup Environ health. 2020;93(7):823-38. <https://doi.org/10.1007/s00420-020-01535-4>
7. Bastien MF, Corbière M. Return-to-Work Following Depression: What Work Accommodations Do Employers and Human Resources Directors Put in Place? J Occup Rehabil. 2019;29(2):423-432. <https://doi.org/10.1007/s10926-018-9801-y>
8. Blank L, Peters J, Pickvance S, Wilford J, MacDonald E. A systematic review of the factors which predict return to work for people suffering episodes of poor mental health. J Occup Rehabil. (2008); 18(1): 27-34. <https://doi.org/10.1007/s10926-008-9121-8>
9. Boštjančič E, Galič K. Returning to work after sick leave–The role of work demands and resources, self-efficacy, and social support. Front Psychol. 2020; 11:661. <https://doi.org/10.3389%2Ffpsyg.2020.00661>
10. Brenninkmeijer V, Lagerveld SE, Blonk RW, Schaufeli WB, Wijngaards-de Meij LD. Predicting the effectiveness of work-focused CBT for common mental disorders: the influence of baseline self-efficacy, depression and anxiety. J. Occup. Rehabil. 2019;29(1):31-41. <https://doi.org/10.1007/s10926-018-9760-3>
11. British Occupational Health Research Foundation (n.d.). Workplace interventions for people with common mental health problems. A summary for employers and employees. <https://www.bohrf.org.uk/downs/cmh_emp.pdf>
12. Björk L, Glise K, Pousette A, Bertilsson M, Holmgren K. Involving the employer to enhance return to work among patients with stress-related mental disorders–study protocol of a cluster randomized controlled trial in Swedish primary health care. BMC Public Health. 2018;18(1):1-9. <https://doi.org/10.1186/s12889-018-5714-0>
13. Corbière M, Mazaniello-Chézol M, Bastien MF, Wathieu E, Bouchard R, Panaccio A, et al. Stakeholders’ role and actions in the return-to-work process of workers on sick-leave due to common mental disorders: a scoping review. J. Occup. Rehabil. 2020;30(3):381-419. <https://doi.org/10.1007/s10926-019-09861-2>
14. Cullen KL, Irvin E, Collie A, Clay F, Gensby U, Jennings PA, et al. Effectiveness of Workplace Interventions in Return-to-Work for Musculoskeletal, Pain-Related and Mental Health Conditions: An Update of the Evidence and Messages for Practitioners. J Occup Rehabil. 2017; 28(1): 1-15. <https://doi.org/10.1007/s10926-016-9690-x>
15. Dalgaard VL, Aschbacher K, Andersen JH, Glasscock DJ, Willert MV, Carstensen O, et al. Return to work after work-related stress: a randomized controlled trial of a work-focused cognitive behavioral intervention. Scand J Work Environ Health, 2017; 43(5): 436-446. <https://doi.org/10.5271/sjweh.3655>
16. Dewa CS, Trojanowski L, Joosen MC, Bonato S. Employer Best Practice Guidelines for the Return to Work of Workers on Mental Disorder–Related Disability Leave: A Systematic Review. Can J Psychiatry. 2016; 61(3), 176-185. <https://doi.org/10.1177/0706743716632515>
17. Durand MJ, Corbière M, Coutu MF, Reinharz D, Albert V. A review of best work-absence management and return-to-work practices for workers with musculoskeletal or common mental disorders. Work. 2014; 48(4): 579-589.
18. Etuknwa A, Daniels K, Eib C. Sustainable return to work: a systematic review focusing on personal and social factors. J. Occup. Rehab. 2019;29(4):679-700. <https://doi.org/10.1007/s10926-019-09832-7>
19. Fischer JE, Genser B, Nauroth P, Litaker D, Mauss D. Estimating the potential reduction in future sickness absence from optimizing group-level psychosocial work characteristics: a prospective, multicenter cohort study in German industrial settings. J Occup Med Toxicol. 2020; 15(1). <https://doi.org/10.1186/s12995-020-00284-x>
20. IRRST. Supporting a Return to Work after an Absence for a Mental Health Problem Design, Implementation, and Evaluation of an Integrated Practices Program. 2014. <http://www.irsst.qc.ca/media/documents/PubIRSST/R-823.pdf>
21. Kenning C, Lovell K, Hann M, Agius R, Bee PE, Chew-Graham C, et al. Collaborative case management to aid return to work after long-term sickness absence: a pilot randomised controlled trial. Public Health Res 2018;6(2). <https://doi.org/10.3310/phr06020>
22. Kouvonen A, Mänty M, Harkko J, Sumanen H, Konttinen H, Lahti J, et al. Effectiveness of internet-delivered cognitive behavioural therapy in reducing sickness absence among young employees with depressive symptoms: study protocol for a large-scale pragmatic randomised controlled trial. BMJ open. 2019;9(10):e032119. <http://dx.doi.org/10.1136/bmjopen-2019-032119>
23. Lokman S, Volker D, Zijlstra-Vlasveld MC, Brouwers EP, Boon B, Beekman AT, et al. Return-to-work intervention versus usual care for sick-listed employees: health-economic investment appraisal alongside a cluster randomised trial. BMJ open. 2017;7(10):e016348. <http://dx.doi.org/10.1136/bmjopen-2017-016348>
24. Mental Health Foundation (n.d). <https://www.mentalhealth.org.uk/publications/returning-work-role-depression>
25. Mikkelsen MB, Rosholm M. Systematic review and meta-analysis of interventions aimed at enhancing return to work for sick-listed workers with common mental disorders, stress-related disorders, somatoform disorders and personality disorders. Occup Environ Med. 2018;75(9):675-86. <http://dx.doi.org/10.1136/oemed-2018-105073>
26. Mind (n.d). Returning to work. <https://www.mind.org.uk/information-support/tips-for-everyday-living/how-to-be-mentally-healthy-at-work/returning-to-work/>
27. Munir F, Yarker J, Hicks B, Donaldson-Feilder E. Returning employees back to work: developing a measure for supervisors to support return to work (SSRW). J Occup Rehabil. 2012; 22(2), 196-208. <https://doi.org/10.1007/s10926-011-9331-3>
28. NICE. Mental wellbeing at work. Public health guideline [PH22]. 2009a. <https://www.nice.org.uk/guidance/ph22>
29. NICE. Workplace health: long-term sickness absence and incapacity to work. Public health guideline [PH19]. 2009b. <https://www.nice.org.uk/guidance/ph19>
30. NICE. Workplace health: long-term sickness absence and capability to work NICE guideline Published: 20 November 2019 [www.nice.org.uk/guidance/ng146](https://lunet-my.sharepoint.com/personal/hufm_lunet_lboro_ac_uk/Documents/My%20Documents/Research/research/Midlands%20Engine/MHPP%207%20Return%20to%20Work%20Project/Phase%201_Completed/Phase%201%20paper/Revised%20paper/www.nice.org.uk/guidance/ng146) [Accessed 22nd October 2020].
31. Pedersen P, Søgaard HJ, Yde BF, et al. Psychoeducation to facilitate return to work in individuals on sick leave and at risk of having a mental disorder: protocol of a randomised controlled trial. BMC Public Health. 2014; 14: 1288. <https://doi.org/10.1186/1471-2458-14-1288>
32. Pomaki G., Franche RL, Murray E, Khushrushahi N, Lampinen TM. Workplace-based work disability prevention interventions for workers with common mental health conditions: a review of the literature. J Occup Rehabil. 2012; 22(2): 182-195. <https://doi.org/10.1007/s10926-011-9338-9>
33. Poulsen R, Fisker J, Hoff A, Hjorthøj C, Eplov LF. Integrated mental health care and vocational rehabilitation to improve return to work rates for people on sick leave because of exhaustion disorder, adjustment disorder, and distress (the Danish IBBIS trial): Study protocol for a randomized controlled trial. Trials. 2017; 18(1):579 <https://doi.org/10.1186/s13063-017-2273-0>
34. Scottish Government. Evaluation of Working Health Services Scotland 2010-2014. 2016. <https://www.gov.scot/Publications/2016/06/6892/downloads>
35. Sikora A, Schneider G, Stegmann R, Wegewitz U. Returning to work after sickness absence due to common mental disorders: study design and baseline findings from an 18 month mixed methods follow-up study in Germany. BMC Public Health. 2019;19(1):1-3. <https://doi.org/10.1186/s12889-019-7999-z>
36. van de Poll MK, Nybergh L, Lornudd C, Hagberg J, Bodin L, Kwak L, et al. Preventing sickness absence among employees with common mental disorders or stress-related symptoms at work: a cluster randomised controlled trial of a problem-solving-based intervention conducted by the Occupational Health Services. Occup Environ Med. 2020;77(7):454-61. <http://dx.doi.org/10.1136/oemed-2019-106353>
37. van Vilsteren M, van Oostrom SH, de Vet HC, Franche RL, Boot CR, Anema JR. Workplace interventions to prevent work disability in workers on sick leave. The Cochrane Library. 2015. <https://doi.org/10.1002/14651858.cd006955.pub3>
38. Victor M, Lau B, Ruud T. Predictors of return to work among patients in treatment for common mental disorders: a pre-post study. BMC Public Health. 2017; 18(1):27. <https://doi.org/10.1186%2Fs12889-017-4581-4>
39. Volker D, Zijlstra-Vlasveld MC, Anema JR, Beekman AT, Brouwers EP, Emons W, et al. Effectiveness of a blended web-based intervention on return to work for sick-listed employees with common mental disorders: results of a cluster randomized controlled trial. J Med Internet Res. 2015; 17(5): e116. <https://doi.org/10.2196/jmir.4097>
40. Wåhlin C, Ekberg K, Persson J, Bernfort L, Öberg B. Association between clinical and work-related interventions and return-to-work for patients with musculoskeletal or mental disorders. J Rehab Med. 2012; 44(4): 355-362. <https://doi.org/10.2340/16501977-0951>
