# Supplementary material for: Using intervention mapping to develop evidence-based toolkits that support workers on long-term sick leave and their managers
Source: BMC Health Serv Res. 2023 Sep 2;23:942. doi: 10.1186/s12913-023-09952-0 (PMC10474744; doi:10.1186/s12913-023-09952-0)
Supplement: Supplementary file 2 — Additional file 2. Performance objectives, determinants and change objectives for the worker on long-term sick leave. list of performance objectives and behaviour change matrix for the worker on long-term sick leave. [file 12913_2023_9952_MOESM2_ESM.docx]

| **Additional file 2:** Performance objectives, determinants and change objectives for the worker on long-term sick leave | | |
| --- | --- | --- |
| **Toolkit** |  |  |
| **Performance Objective** | **Determinants** | **Change Objective(s)** |
| PO.1. Worker decides to use the toolkit | 1a. Intention  1b. Outcome expectation | 1a. Formulate and implement commitment to use toolkit  1b. Expect that using the toolkit will improve wellbeing and relationship with the workplace |
| 1.1 Reviews key information provided in the RTW toolkit about its purpose and benefits | 1.1a Knowledge  1.1b. Perceived norms | 1.1a. Show understanding of toolkit purpose and how to use it  1.1b. Recognise many workers on long-term sick leave may have poor mental wellbeing or experience it whilst on sick leave |
| *1.2* Identifies which step of the toolkit they should start at | 1.2a. Self- efficacy  1.2b. Skills | 1.2a. Demonstrate confidence in using the toolkit  1.2b. Demonstrate ability to assess own confidence and readiness to return to work |
| *Step 1* |  |  |
| PO.2. Worker uses toolkit (wellbeing, work procedures, manager support and contact) and identifies actions to complete from each checklist, if necessary | 2a. Intention  2b. Self- efficacy  2c. Knowledge  2d. Skills  2e. Perceived norms  2f. Outcome expectations | 2a. Demonstrate motivation to monitor and undertake actions  2b. Feel confident in being able to monitor and take action for own wellbeing and support needs  2c. List available workplace policies and procedures related to sick leave. List appropriate actions to take and their benefits  2d. Demonstrate ability to undertake actions identified  2e. Recognise that nowadays workers are being encouraged to take an active part in their care  2f. Expect that using toolkit and undertaking actions will improve own wellbeing and access to support |
| PO2.1 Worker communicates with their manager about their sick leave, return to work intentions (if known), their health and wellbeing (repeated at step 2 and 3) | 2.1a. Intention  2.1b. Self-efficacy  2.1c. Knowledge  2.1d. Skills  2.1e. Outcome expectations | 2.2a. Demonstrate motivation and commitment to communicate with manager  2.2b. Express confidence in in ability to communicate thoughts, feelings and situation with manager  2.2c. Describe understanding of workplace policies and procedures related to sick leave and return to work  2.2d. Demonstrate ability to communicate needs in an open, friendly manner  2.2e. Expect that communicating with workplace will lead to a positive experience with the manager |
| PO2.2 Communicates with their manager about what could help them whilst on sick leave (e.g., what to tell colleagues) | 2.2a. Intention  2.2b. Self-efficacy  2.2c. Knowledge  2.2d. Skills  2.2e. Outcome expectations | 2.3a. Demonstrate motivation and commitment to communicate with manager  2.3b. Express confidence in ability to communicate thoughts, feelings and situation with manager  2.3c. Describe resources and support needs whilst on sick leave  2.3d. Demonstrate ability to communicate in an open, friendly manner  2.3e. Expect that communicating with workplace will lead to support from manager |
| PO.3 Worker identifies any negative thoughts toward communication and/or returning to work (can repeated in step 2 and 3) | 3a. Intention  3b. Self-efficacy  3c. Awareness  3d. Perceived barriers | 3a. Demonstrate motivation and commitment to explore any negative thoughts  3b. Express confidence in in ability to identify negative thoughts  3c. Acknowledge the presence of unhelpful thoughts and feelings  3d. Anticipate negative consequences for unhelpful thoughts and feelings |
| PO3.1 Assess the evidence for any negative thoughts and recognise alternative ways to think about the issue and practices alternative thinking | 3.1a. Framing/reframing  3.1b. Self-efficacy  3.1c. Skills  3.1d. Self-regulation  3.1e. Attitude and emotions  3.1f. Outcome expectations | 3.1a. Recognise the advantage of changing thinking  3.1b. Express confidence in ability to create alternative thoughts  3.1c. Demonstrate ability to re-evaluate thoughts  3.1a. Monitor thoughts and feelings regularly using the checklist  3.1e. Feel positive about re-evaluating thoughts and returning to reframing if necessary  3.1f. Expect that alternative thoughts will lead to more effective relations with manager and improve wellbeing |
| PO4 Worker evaluates their support network (can be repeated in step 2 and 3) | 4a. Intention  4b. Self-efficacy  4c. Knowledge  4d. Behavioural capabilities | 4a. Demonstrate motivation and commitment to evaluate their support network  4b. Express confidence in ability to evaluate the support they have and what support they need  4c. Identifies key persons who could provide support  4d. Actions support by asking relevant person for support |
| PO5 Worker makes the decision to set goals to look after their health and wellbeing (can also be used in step 2 and 3) | 5a. Intention  5b. Skills  5c. Perceived barriers  5d. Knowledge  5e. Behavioural capabilities  5f. Outcome expectations | 5a. Demonstrate motivation and commitment to set goals  5b. Demonstrate how to set a goal  5c. Anticipate own barriers and environmental barriers to achieving the gaol  5d. Describe the steps to take (actions) to reach the goal  5e. Undertake the actions to achieve the set goal  5f. Expect that achieving the goal will improve physical and mental wellbeing |
| *Step 2: Preparing to return to work* |  |  |
| PO6. Worker is ready to RTW and identifies the demands of their work and what work adjustments might be useful for returning to work | 6a. Intention  6b. Skills  6c. Self-efficacy  6d. Knowledge  6e. Perceived norms  6f. Outcome expectations | 6a. Demonstrate motivation and commitment to returning to work  6b. Demonstrate ability to assess own confidence and readiness to return to work  6c. Express confidence in completing their ability to prepare returning to work  6d. Describe the demands of work and the work adjustments that would help in return to work, and to stay in work  6e. Recognise that managers are responsible for supporting RTW outcomes and talking to them at an early stage can facilitate RTW expectations  6f. Expects that engaging with the manager and other relevant stakeholders on their RTW plan will lead to a positive RTW outcome |
| PO6.1 Develops action plan for discussing RTW and potential work adjustments | 6.1a. Knowledge  6.1b. Perceived norms | 6.1a. Lists the potential barriers and solutions to their own RTW and develops a RTW plan  6.1b. Recognise that managers do not necessarily know all the tasks workers undertake, all the details of workers’ health and wellbeing, and the impact of work on health and vice versa |
| PO6.2 Discusses RTW date, plan and potential work adjustments using checklists and action plan with manager | 6.2a. Self-efficacy  6.2b. Skills  6.2c. Attitudes and emotions  6.2d. Outcome expectations | 6.2a. Express confidence in communicating with manager about their RTW plans  6.2b. Demonstrate how to negotiate a RTW plan with their manager; communicates in an open and friendly manner  6.2c. Feel positive about returning to work  6.2d. Expects that identifying and prioritising potential barriers and facilitators to RTW, and developing a RTW plan, with employer as required, will lead to a safe and appropriate RTW |
| *Step 3: being back at work* |  |  |
| PO7 Monitors wellbeing and work performance, work adjustments, support and wellbeing | 7a. Self-efficacy  7b. Self-regulation  7c. Attitudes and emotions | 7a. Express confidence in ability to monitor wellbeing and performance  7b. Monitor wellbeing and performance regularly  7c. Feel positive about being back at work |
| PO7.1 Reviews barriers and facilitators to wellbeing and performance and takes action to address barriers | 7.1a. Self-efficacy  7.1b. Knowledge  7.1c. Behavioural capabilities  7.1d. Outcome expectations | 7.1a. Express confidence in identifying barriers/facilitators to their own wellbeing and work performance  7.1b. Lists the potential barriers and solutions to their own wellbeing and performance  7.1c. Develop a plan with the manager as required  7.1d. Expects that identifying and prioritising potential barriers and facilitators to wellbeing and performance, and developing plan, with manager as required, will lead to an increase in wellbeing and work performance |
