# Supplementary material for: Using intervention mapping to develop evidence-based toolkits that support workers on long-term sick leave and their managers
Source: BMC Health Serv Res. 2023 Sep 2;23:942. doi: 10.1186/s12913-023-09952-0 (PMC10474744; doi:10.1186/s12913-023-09952-0)
Supplement: Supplementary file 3 — Additional file 3. Performance objectives, determinants and change objectives for the workplace health coach. List of performance objectives and behaviour change matrix for the workplace coach. [file 12913_2023_9952_MOESM3_ESM.docx]

| **Additional file 3:** Performance objectives, determinants and change objectives for the workplace health coach | | |
| --- | --- | --- |
| **Performance Objective** | **Determinants** | **Change Objective(s)** |
| PO1. Workplace health coach encourages worker to use the RTW toolkit | 1a. Attitude  1b. Self-efficacy  1c. Knowledge | 1a. Express feelings about the benefits of the RTW toolkit  1b. Express confidence that they can motivate the worker to use the toolkit during their sick leave and return to work  1c. State the benefits of using the toolkit |
| PO2. Workplace health coach helps worker to identify and set goals to support wellbeing | 2a. Attitude  2b. Self-efficacy  2c. Skills  2d. Social influence | 1a. Express feelings about the benefits of identifying and setting goals and the actions to reach the goal  2b. Express confidence in actively listening to worker and confidence in helping worker to identify appropriate goals and actions  2c Demonstrate actively listening to worker’s concerns about sick leave and ideas around goal setting  2d. Encourage the worker to under the actions to reach their goal |
| PO3. Workplace health coach supports the worker in preparing to RTW | 3a. Attitude  3b. Self-efficacy  3c. Skills  3d. Social influence | 1a. Express feelings about the benefits of planning return to work barriers and facilitators before meeting with the manager  2b. Express confidence in actively listening to worker’s concerns about returning to work and confidence in helping worker to identify appropriate goals and actions  2c Demonstrate actively listening to worker’s concerns and ideas around goal setting  2d. Encourage the worker to under the actions to reach their goal |
