# Supplementary material for: Using intervention mapping to develop evidence-based toolkits that support workers on long-term sick leave and their managers
Source: BMC Health Serv Res. 2023 Sep 2;23:942. doi: 10.1186/s12913-023-09952-0 (PMC10474744; doi:10.1186/s12913-023-09952-0)
Supplement: Supplementary file 4 — Additional file 4. Performance objectives, determinants and change objectives for the manager. List of performance objectives and behaviour change matrix for the manager. [file 12913_2023_9952_MOESM4_ESM.docx]

| **Additional file 4**: Performance objectives, determinants and change objectives for the manager | | |
| --- | --- | --- |
| **Toolkit** |  |  |
| **Performance Objective** | **Determinants** | **Change Objective(s)** |
| PO1. Manager uses the toolkit | 1a. Intention  1b. Knowledge  1c. Self- efficacy  1d. Skills  1e. Perceived norms  1f. Outcome expectation | 1a. Formulate and implement commitment to use toolkit for managing workers’ long-term sickness absence and return to work  1b. Describe purpose of the toolkit to the worker and how and when to use it  1c. Express confidence in monitoring and undertaking actions outlined in the toolkit  1d. Demonstrate ability to undertake and record appropriate actions in each step if the toolkit  1e. Recognise many workers on long-term sick leave may have poor mental wellbeing or experience it whilst on sick leave  1f. Expect that using the toolkit will help manager deliver positive experience to the worker through good communication and support |
| *Step 1* |  |  |
| PO2. Manager manages initial sick leave by contacting the worker to explain sick leave policy and procedures and the support they can offer the worker whilst on sick leave | 2a. Attitude  2b. Knowledge  2c. Self- efficacy  2d. Skills  2e. Outcome expectations | 2a. Express positive feelings toward managing initial long-term sick leave  2b. Describe ways to express support to the worker on sick leave  2c. Express confidence in contacting the worker on sick leave  2d. Demonstrate ability to communicate with the worker in an open, friendly manner  2e. Expect that communicating with worker early on will lead to a positive experience for the worker |
| PO2.1. Keeps in regular contact with worker’s during their sick leave | 2.1a. Attitude  2.1b. Knowledge  2.1c. Self- efficacy  2.1d. Skills  2.1e. Social Influence  2.1f. Outcome expectations | 2.1a. Express positive feelings about keeping in regular touch with the worker during their sick leave  2.1b. List how, when and how often they will keep in touch with the worker  2.1c. Express confidence in keeping in regular touch with the worker  2.1d. Demonstrate ability to communicate regularly with the worker  2.1e. Encourage the worker to keep in regular touch  2.1f. Expect that communicating with worker regularly will lead to a positive experience for the worker |
| *Step 2: Preparing worker to return to work* |  |  |
| PO3. Manager follows the toolkit procedures for preparing the worker to return to work | 3a. Knowledge  3b. Self-efficacy  3c. Skills  3d. Awareness  3e. Perceived norms  3f. Outcome expectations | 3a. Explain the importance of following best practice procedures to support the worker in returning to work  3b. Express confidence that they can support the work in their return  3c. Demonstrate ability to follow best practice procedures for supporting RTW  3d. Acknowledge the need to change environmental cues to support the return to work outcome  3e. Recognise that managers are responsible for supporting RTW outcomes and talking to the worker about their return at an early stage can facilitate RTW expectations  3f. Expect that involving the worker on their RTW plan will lead to a positive RTW outcome |
| PO3.1 Agrees RTW date, plan and potential work adjustments with the worker using best practice guidelines | 3.1a Knowledge  3.1b. Self-efficacy  3.1c. Skills  3.1d. Social Influence  3.1e. Outcome expectations | 3.1a. List RTW best practice procedures including appropriate work adjustments  3.1b. Express confidence in communicating with worker about their RTW plans  3.1c. Demonstrate ability to create a joint RTW plan with their worker by communicating in an open and friendly manner  3.1d. Indicate that they plan to support the return of the worker  3.1e. Expect developing a RTW plan with the worker will lead to a positive and sustainable RTW |
| *Step 3: Supporting the worker back at work* |  |  |
| PO4. Manager supports worker on their first day back and checks-in on their wellbeing and work adjustments | 4a. Knowledge  4b. Self-efficacy  4c. Skills  4d. Behavioural capabilities  4e. Outcome expectations | 4a. Lists the potential supports and additional adjustments worker might need on their return to work  4b. Express confidence in ability to support worker on their first day back at work  4c. Demonstrate ability to provide appropriate support to worker on their first day back  4d. Develop a plan with the manager as required  4e. Expect that supporting worker on their first day back will contribute to a sustainable RTW |
| PO4.1 Manager regularly meets with worker to check-in on their wellbeing, adjustments and work performance | 4.1a. Self-efficacy  4.1b. Skills  4.1c. Self-regulation  4.1d. Social influence  4.1e. Outcome expectation | 4.1a. Express confidence in ability to monitor worker’s wellbeing and performance  4.1b. Demonstrate ability to ask worker about their wellbeing and performance in a positive manner  4.1c. Monitor worker’s wellbeing and performance regularly  4.1d. Encourage the worker to discuss their wellbeing and performance  4.1e. Expect that regular check-in with the worker will contribute to a sustainable RTW |
