# Supplementary material for: Using intervention mapping to develop evidence-based toolkits that support workers on long-term sick leave and their managers
Source: BMC Health Serv Res. 2023 Sep 2;23:942. doi: 10.1186/s12913-023-09952-0 (PMC10474744; doi:10.1186/s12913-023-09952-0)
Supplement: Supplementary file 5 — Additional file 5. Performance objectives, determinants and change objectives for the Human resources team. List of performance objectives and behaviour change matrix for the manager. [file 12913_2023_9952_MOESM5_ESM.docx]

| **Additional file 5:** Performance objectives, determinants and change objectives for the Human resources team | | |
| --- | --- | --- |
| **Performance Objective** | **Determinants** | **Change Objective(s)** |
| PO1. Communicate the new toolkits across the organisation | 1a. Knowledge  1b. Skills  1c. Social influence  1d. Outcome expectations | 1a. List the different strategies to communicate about the study and the toolkits  1b. Demonstrate ability to implement a monthly communication strategy  1c. Indicate the benefits of the toolkit  1d. Expect that regular communication will contribute to the use of the toolkits |
| PO2. Informs workers on long-term sick leave and their manager that they will receive a RTW toolkit to use and why | 1a. Knowledge  1b. Self-efficacy  1c. Skills  1d. Social influence  1e. Outcome expectations | 1a. List the different steps for the worker on long term sick leave and their manager to join PROWORK.  1b. Express confidence in ability to engage with managers and workers  1c. Demonstrate ability to support managers and workers  1d. Encourage managers and workers to use the toolkits  1e. Expect that contacting the managers and workers directly will contribute to the use of the toolkits |
