# Supplementary material for: Using intervention mapping to develop evidence-based toolkits that support workers on long-term sick leave and their managers
Source: BMC Health Serv Res. 2023 Sep 2;23:942. doi: 10.1186/s12913-023-09952-0 (PMC10474744; doi:10.1186/s12913-023-09952-0)
Supplement: Supplementary file 6 — Additional file 6. Summary of selected theories. It includes a description of the theoretical change methods to change behaviour from our stated change objectives and a summary of the theories and their determinants. [file 12913_2023_9952_MOESM6_ESM.docx]

| **Additional file 6:** Summary of selected theories | | |
| --- | --- | --- |
| **Theory** | **Determinants from table Xx** | **Summary** |
| Communication Accommodation Theory (CAT) (Giles, 1973; Gallios et al, 2005) | Intention  Knowledge  Skills  Social influence | Theoretical framework of interpersonal and intergroup communication. Aims to understand and anticipate why, when, and how people adjust their communicative behaviour according to situations and people involved; and what social consequences result from those adjustments. |
| Implementation Intentions (Gollwitzer, 1999) | Intentions  Skills  Self-regulation | Describe a self-regulatory strategy that involves an individual planning ahead (e.g. when, where and how), the situation in which they will perform a particular behaviour. |
| Conservation of Resources (CoR) Theory (Hobfoll, 1989) | Knowledge  Skills  Awareness  Social influence | Describes the motivation of people to maintain and protect resources, recover from resource loss, and gain resources in the face of stress. Resources can be personal (e.g., self-esteem, health, coping, locus of control), social (e.g., support from manager and colleagues), organisational (e.g., resources that help the individual to do their job, achieve work goals or stimulate personal growth), and environmental (e.g., sick pay, transport). |
| Transtheoretical Model of Change (Prochaska and DiClemente, 1982 | Knowledge  Skills  Behavioural capabilities | Model suggests that health behaviour change involves progress through six stages of change: precontemplation, contemplation, preparation, action and maintenance. At any stage, it can also lead to termination of the behaviour |
| Socio-Cognitive Theory (SCT) (Bandura, 1986). | Knowledge  Self-efficacy  Self-regulation  Outcome expectations | Suggests the environment (e.g., resources, support from family/friends), behaviour, personal factors (e.g., age, previous experience with the behaviour) and cognitive factors (e.g., conceptions, observations, and motivation) all interact to influence each other. The theory outlines key capabilities to further explain the interactions: self-regulatory capability refers to the individual’s ability to modify their own behaviour by examining personal standards against actual behaviour and making subsequent adjustments. Self-efficacy, a type of reflective capability, refers to the individual’s belief in their capacity or ability to undertake a behaviour. |
| Cognitive theory (Beck, 1976; Ellis, 1962). | Attitudes and emotions  Framing/reframing  Self-regulation | Posits that the way people hold assumptions about themselves and perceive their experiences influences their emotional, behavioural, and physiological reactions. Correcting misperceptions and modifying unhelpful thinking and behaviour brings about improved reactions. |
